# Surface-enhanced mid-infrared absorption spectroscopy using miniaturized-disc metasurface

**DOI:** 10.1038/s41598-021-02984-8

**Published:** 2021-12-07

**Authors:** Mitchell Semple, Ashwin K. Iyer

**Affiliations:** Department of Electrical and Computer Engineering, University of Alberta, Edmonton, Alberta Canada

**Keywords:** Mid-infrared photonics, Sub-wavelength optics, Nanophotonics and plasmonics, Metamaterials

## Abstract

Surface-enhanced infrared spectroscopy is an important technique for improving the signal-to-noise ratio of spectroscopic material identification measurements in the mid-infrared fingerprinting region. However, the lower bound of the fingerprinting region receives much less attention due to a scarcity of transparent materials, more expensive sources, and weaker plasmonic effects. In this paper, we present a miniaturized metasurface unit cell for surface-enhanced infrared spectroscopy of the 15-$\upmu$m vibrational band of CO$_{2}$. The unit cell consists of a gold disc, patterned along the edge with fine gaps/wires to create a resonant metamaterial liner. In simulation, our plasmonic metamaterial-lined disc achieves greater than $4\times$ the average field intensity enhancement of a comparable dipole array and a miniaturized size of $\lambda _0/5$ using complex, 100-nm features that are patterned using 100-kV electron-beam lithography. In a simple experiment, the metamaterial-lined disc metasurface shows a high tolerance to fabrication imperfections and enhances the absorption of CO$_{2}$ at 15 $\upmu$m. The resonant wavelength and reflection magnitude can be tuned over a wide range by adjusting the liner feature sizes and the metasurface array pitch to target other vibrational bands. This work is a step toward low-cost, more compact on-chip integrated gas sensors.

## Introduction

Metasurfaces (MTSs) have garnered significant interest due to their ability to manipulate electromagnetic waves on a subwavelength scale. MTSs are periodic or quasiperiodic 2D arrays composed of subwavelength meta-atoms, where each meta-atom is tailored to exhibit a specific electromagnetic response. With the proper implementation, MTSs have been designed for a variety of applications, including beam redirection at arbitrary angles, polarization conversion, perfect absorption, and for other more complex wavefront transformations such as holograms and Bessel beams^1,2^. Furthermore, when placed in the near-field of a sample under test, MTSs have been realized for subdiffraction imaging and magnification, material sensing, and surface-enhanced spectroscopy^3–7^.

Surface-enhanced spectroscopy is a well-known and widely used technique for nondestructive chemical analysis and imaging. Typically, the material under test is loaded onto a metallic (plasmonic) surface and the metal is excited at its surface plasmon resonance frequency. This induces a strong local electric field (*E*), which enhances a particular Raman scattering or absorption line. Depending on sample preparation, Raman scattering efficiency can be improved by a factor of $>|E|^4$, whereas absorption can be improved by a factor of $\sim |E|^2$^8,9^. In fact, in the case of Raman scattering, even single molecules have been detected using arrays of resonant plasmonic metal nanoparticles with small inter-particle gaps to excite strong electric-field “hotspots”. Although a number of fundamental molecular vibrational modes are exclusively Raman active, i.e., requiring a shift in the polarizability of the molecule, other molecular vibrations are exclusively IR active, i.e., requiring a shift in the dipole moment of the molecule (in symmetric molecules, these categories are mutually exclusive). Surface-enhanced infrared absorption (SEIRA) spectroscopy is hence required for complementary characterization of a sample’s vibrational spectrum. In particular, gases are difficult to characterize using SEIRA spectroscopy due to their low density and variable adsorption rates. Instead, bulky and expensive non-dispersive IR (NDIR) absorption methods that rely on transmission through a gas sample are generally used. Despite the reliability of NDIR, increasing the sensitivity SEIRA spectroscopy to gases by optimizing the hotspots or adding functionalization layers would decrease the cost and required size, increase the selectivity, and create miniaturized on-chip real-time gas sensors. SEIRA spectroscopy generally targets the chemical fingerprinting region of the IR spectrum between 1500 and 500 cm$^{-1}$ (6.67–20 $\upmu$m wavelength), where every material has a unique absorption band fingerprint controlled by photons exciting higher-order vibrational states in the molecular structure.

A wide variety of materials, fabrication methods, and nanoparticle shapes have been developed to improve the signal enhancement in SEIRA^10–19^; however, long-wavelength SEIRA (beyond $\sim$700 cm$^{-1}$) has developed more slowly due to the scarcity of transparent substrate materials, lower signal levels, more costly sources/detectors, and difficulties making use of plasmonic resonant nanoparticle field enhancement at these wavelengths. The natural plasmonic effect is unable to miniaturize resonators at these wavelengths, which leads to large inter-particle distances and a low density of electric-field hotspots. An ideal SEIRA surface would be made of miniaturized, subwavelength elements that are closely spaced, which allows them to exhibit a high density of hotspots across the sample surface for maximum efficiency in a similar manner to colloidal metal surfaces^20^. Nevertheless, the capacitive and plasmonic field enhancement between resonant nanoantenna tips has been successfully used to significantly enhance SEIRA signals at these hotspots^21–23^. Commonly used dipole-nanoantenna arrays require creating large, resonant elements (on the order of $\lambda _0/4$ or larger) that exhibit a mere two hotspots per period, making inefficient use of the illuminated sample area. Dipole-nanoantenna arrays also respond only to electric fields polarized along their axis, requiring the addition of polarizing elements to the measurement setup and only enhancing the absorption of aligned molecules. One approach to address this challenge has been to use cross-shaped dipoles instead, which allows any randomly oriented molecule to be excited, but the elements still exhibit only four hotspots per period^13^.

Meta-atoms are generally made of subwavelength (hence miniaturized) metal or dielectric resonators that predictably scatter incoming light and hence offer new techniques for designing SEIRA arrays. Dielectric, multilayer, and encased plasmonic resonant meta-atoms exhibit strongly enhanced fields inside the dielectric, making the enhanced fields inaccessible for sensing the vibrational modes of nearby materials. Conversely, metallic resonant meta-atoms store energy outside the structure where the fields are accessible. Using the design principles of artificial plasmas and transmission-line metamaterials (MTMs)^24,25^, meta-atoms can be made to exhibit surface-plasmon-like resonances at much longer wavelengths than normally possible, improving the yield of SEIRA signals while the fields continue to be accessible.

Recently, meta-atoms based on metallic discs and apertures lined with MTMs have been developed for applications from the microwave regime to the visible regime^7,26–29^. The MTM-lined aperture, and its dual, the MTM-lined disc, exhibit plasmon-like resonances that are ideal for SEIRA applications as they have been developed using single-layer fabrication technologies, are highly miniaturized at resonance, can be densely packed, and exhibit strong resonant field enhancement in the liner region throughout the electromagnetic spectrum. Scaling the MTM-lined meta-atoms to operate in the fingerprinting region while preserving their strong field enhancement and miniaturization may significantly improve the sensitivity of SEIRA measurements with a marginal increase in complexity. This could allow miniaturization of SEIRA spectroscopic devices and require less sample material for accurate analysis.

CO$_{2}$ detection is a critical step in carbon capture systems for reducing air pollution, and is important in measuring air quality, testing lung function, and quantifying industrial and automotive emissions. CO$_{2}$ is usually sensed near 4.2 $\upmu$m, however, this wavelength range has many other confounding species often found in exhaust plumes, such as H$_{2}$O, O$_{3}$, and CO, that may diminish sensitivity when wavelength resolution or the concentration is low^30–32^. Instead, we target the strong 15-$\upmu$m bending-vibration absorption band responsible for the majority of atmospheric heating by CO$_{2}$, which is useful in multispectral sensing to better discriminate between CO$_{2}$ and other materials. This wavelength range can also be used individually to distinguish aromatic isomers, such as xylenes^33^.

In this paper, we aim to scale the MTM-lined disc MTS to operate at the lower edge of the fingerprinting region, where SEIRA surfaces are typically unable to generate large average electric-field intensity enhancement. Our scaling approach relies on combining aspects of the microwave and optical designs of the MTM-lined aperture/disc to achieve a resonance that can be scaled to any wavelength by appropriately balancing the design aspects. Our design approaches the ideal SEIRA surface condition by making use of long, meandered gaps with thin wires to create strongly enhanced fields over the entire unit cell area. The miniaturization is especially important for these longer wavelengths, where the unit-cell size necessarily increases, which may lead to low filling factors and hence low average field intensity enhancement over the unit-cell. The circular shape of the unit cell allows the resonance to be excited by any input polarization, which simplifies the measurement instrumentation by not requiring polarizers or polarization alignment, and can be easily fabricated over large areas, which allows excitation by poorly focused beams. Moreover, the simple fabrication process can easily be extended to include CMOS processes or functionalization layers. We have previously shown the fabrication and characterization of the nominal MTS array^34^.

To avoid a functionalization layer that would add unnecessary complication to the proof-of-concept, such as thermal regeneration cycles and additional fabrication steps, we validate our approach using a simple CO$_{2}$ gas cell that produces a reflection contrast with low sensitivity but is easily compared to simulation. Having verified our results experimentally, we conclude by suggesting how a thin functionalization layer, modeled as a uniform, thin layer of CO$_{2}$, would improve the sensing performance of the MTS and how the MTM-lined disc can be scaled to target other material resonances in the future.

**Figure 1:**
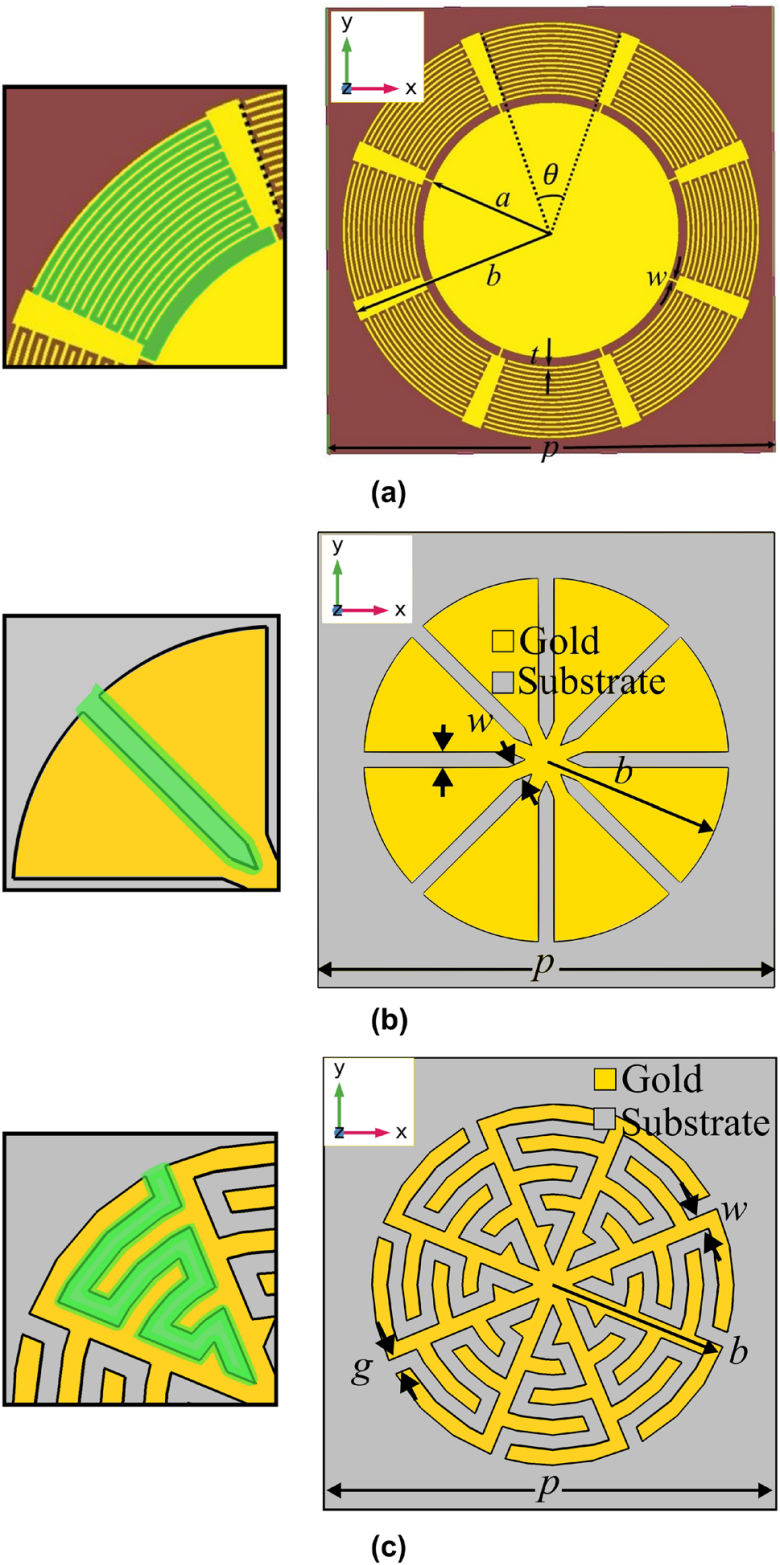
(**a**) Microwave MTM-lined disc, where $\theta$ is the angular interdigitated capacitor span, *a* is the radius of the inner solid disc, *b* is the disc radius including the liner, *w* is the inductive connecting trace width, *t* is the capacitor trace width, and *p* is the array pitch^26^. (**b**) NIR MTM-lined disc, where *b* is the disc radius, *w* is the capacitive gap width and minimum central nanowire width, and *p* is the array pitch. (**c**) MIR MTM-lined disc and the subject of the present paper, where *b* is the total disc radius, *w* is the nanowire width throughout the unit cell, *g* is the nanogap width throughout the unit cell, and *p* is the array pitch^35^. Each panel includes an inset with the capacitive gaps highlighted in green . (**a**) Licensed under CC BY 4.0, modified with axis and highlighted gap insets^26^, (**c**) ©IEEE, modified with permission^35^.

## Results and discussion

### Scaling the MTM liner to the MIR

Performing SEIRA spectroscopy in the fingerprinting region of the spectrum could lead to miniaturization of spectroscopic devices, more sensitive detection of lower concentration materials, and smaller required sample sizes. Since the SEIRA absorption signal is proportional to the resonant electric-field intensity, the goal is to create maximum average field intensity enhancement over the MTS area. Baladi et al. found that when the perimeter of a circular metallic disc is lined with a transmission-line MTM consisting of strong series capacitive loading and weak parallel inductive loading, plasmon-like resonant reflection can be induced well below the usual fundamental resonance frequency of the patch^26^. Due to the miniaturized size at resonance, the liner region strongly confines and enhances the local electric fields, which causes the resonance frequency and reflectance to become very sensitive to nearby materials. Such resonances have also been observed in the MIR and near-IR domains^28,35^.

When designed to operate in the microwave regime, the MTM-lined disc, shown in Fig. 1a, consists of a central solid disc (radius *a*) surrounded by an MTM region ($a<r<b$) and resonates at a frequency of 2.4 GHz (12.5 cm wavelength, $p=1.8$ cm unit cell size). At resonance, the interdigitated capacitors loading the liner region (highlighted in green) exhibit strong field enhancement, and the resonant frequency can be tuned by changing the geometrical parameters of the capacitors ($\theta$, *t*). Eight capacitors are arranged azimuthally for polarization isotropy. When scaled to the near-IR, interdigitated capacitors cannot be used due to the small scale (on the order of $p=300$ nm). Instead, the natural plasmonic response of the metal can be harnessed, and strong capacitive coupling can be imparted with simple straight capacitive nanogaps (highlighted green), as shown in Fig. 1b. This also requires expanding the liner region to encompass nearly the full disc radius and using high-precision 10-nm features, but maintains the strong electric-field enhancement in the liner region.

To target the MIR for the present design, we combine aspects of the near-IR and the microwave MTM-lined discs by using a capacitively loaded plasmonic liner. We begin with the NIR MTM-lined disc, which was designed to resonate at a wavelength of 1.55 $\upmu$m. Targeting the MIR CO$_{2}$ absorption band of 15 $\upmu$m, we scale the size of the structure by a factor of $\sim$10. This scaling maintains the thick liner region, but scales the gap size and disc radius. As the disc grows, the resonance wavelength would normally increase linearly, however, due to material dispersion and the weaker miniaturization of the plasmonic effect, the 10$\times$ scaling does not tune the resonance wavelength directly to 15.5 $\upmu$m. Hence, to tune the resonance down to 15 $\upmu$m and miniaturize the resonant unit-cell further, we interdigitate the capacitive gaps, like in the microwave design. The number of interdigitations can be used to tune the gap capacitance and thus the resonant wavelength of the unit cell. This interdigitation also allows the field enhancement in the capacitive gaps to be spread out over the full unit-cell area, increasing its uniformity. Figure 1c depicts the nominal design, with an interdigitated capacitor highlighted in green, and with geometric parameters as described below.

Gold is used for the metallic layer as it is a good conductor in the MIR and is chemically stable, and ZnSe is used as a substrate since it is one of few crystals that are transparent at this wavelength and insoluble in water. We apply the $\sim$10$\times$ scaling to many of the geometric features to create the nominal design. The film thickness is held at $t=50$ nm for ease of fabrication. The minimum feature size *w* is scaled from 10 nm to 100 nm, which decides the fabrication method. Although the NIR design required high-precision helium focused-ion-beam milling, we chose 100-kV electron-beam lithography (EBL) for the MIR design as it can pattern a resist with features as small as 20 nm and has a much higher throughput. Additionally, EBL is better-suited to creating arrays of disconnected patches than creating arrays of apertures, hence the choice of MTM-lined discs over MTM-lined apertures. Next, the overall size of the unit cell is scaled by a factor of 10, resulting in a $p=3$ $\upmu$m pitch and a $b=1.2$ $\upmu$m patch radius. Finally, the straight nanogaps are meandered to tune the resonance wavelength to 15 $\upmu$m. We continue to use eight azimuthal cells for polarization isotropy.

**Figure 2:**
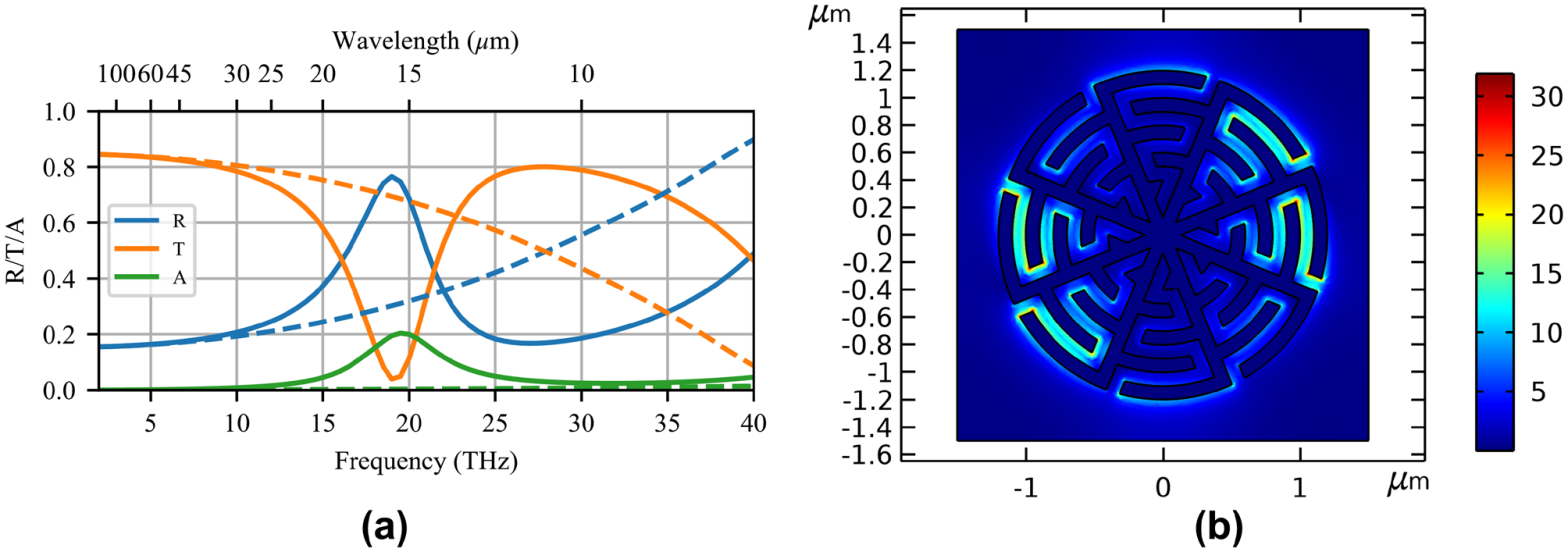
(**a**) Reflection, transmission, and absorption spectra for the nominal MTM-lined MTS structure (solid) and an equivalent unlined-disc array (dashed). (**b**) Electric-field enhancement ratio for the nominal MTM-lined MTS at the resonance wavelength of 15 $\upmu$m. The nominal structure has $b=1.2$ $\upmu$m, $p=3$ $\upmu$m, and $g=w=100$ nm, ©IEEE, modified with permission to add scale bars to part **(**b)^35^.

To validate our approach, we have previously shown^35^ the nominal structure depicted in Fig. 1c, simulated in COMSOL Multiphysics. The results are reproduced in Fig. 2a (solid curves), compared to an equivalent array of solid discs with equal radius *b* (dashed curves). Subject to the minimum feature size, 5 capacitive teeth (equivalently, 5 meanders of the gap) are required to tune the resonance to the desired 15-$\upmu$m (20-THz) wavelength. The resonance profile exhibits an enhancement in reflection coefficient from 0.30 to 0.77 and a distinct Fano-shape, which allows increased contrast when an absorbing species is present and reduces the resonance bandwidth to better target the desired absorption line. Outside the resonant band, the MTM-lined MTS follows a similar trend to that of the unlined disc array. The unlined disc array reflectance increases toward a resonance above 40 THz, which shows that the MTM loading is indeed able to miniaturize the discs, and that no structural resonances unrelated to the MTM loading occur in the active spectral region. Figure 2b shows the ratio of the input electric-field magnitude to the resonant electric-field magnitude (i.e. electric-field enhancement) at 15 $\upmu$m/20 THz for a horizontally polarized excitation. Electric fields are enhanced over the entire liner region with a peak enhancement ratio greater than 30. The field enhancement would be more uniform for an unpolarized excitation due to the polarization symmetry of the cell. Since this peak value may be subject to numerical inaccuracies and SEIRA signal enhancement is proportional to the electric-field *intensity* enhancement, a more meaningful measure of the field enhancement is the average electric field intensity enhancement factor, $F_I$, of 24.5, averaged over the full unit-cell area and film thickness. This is in contrast to the prevailing approach in the literature, which is to report the average intensity enhancement near a hotspot, creating ambiguity in the required averaging volume^8^. Although a solid circular disc array is a good comparison for miniaturization and to show the creation of a new resonance, $F_I$ cannot be similarly compared as fewer degrees of freedom prevent tuning the unlined disc resonance to 15 $\upmu$m with an equivalent footprint. To create a meaningful comparison using the full average intensity metric, we have designed an equivalent conventional coupled-dipole array resonating at 15 $\upmu$m and with equal feature sizes and the same unit-cell area (see Methods for more information). The coupled-dipole array design has a maximum field enhancement ratio of 44 and an $F_I$ of 5.2, less than one quarter that of the MTM-lined disc. Additionally, the resonance is significantly less selective than the MTM-lined disc.

### Field enhancement

**Figure 3:**
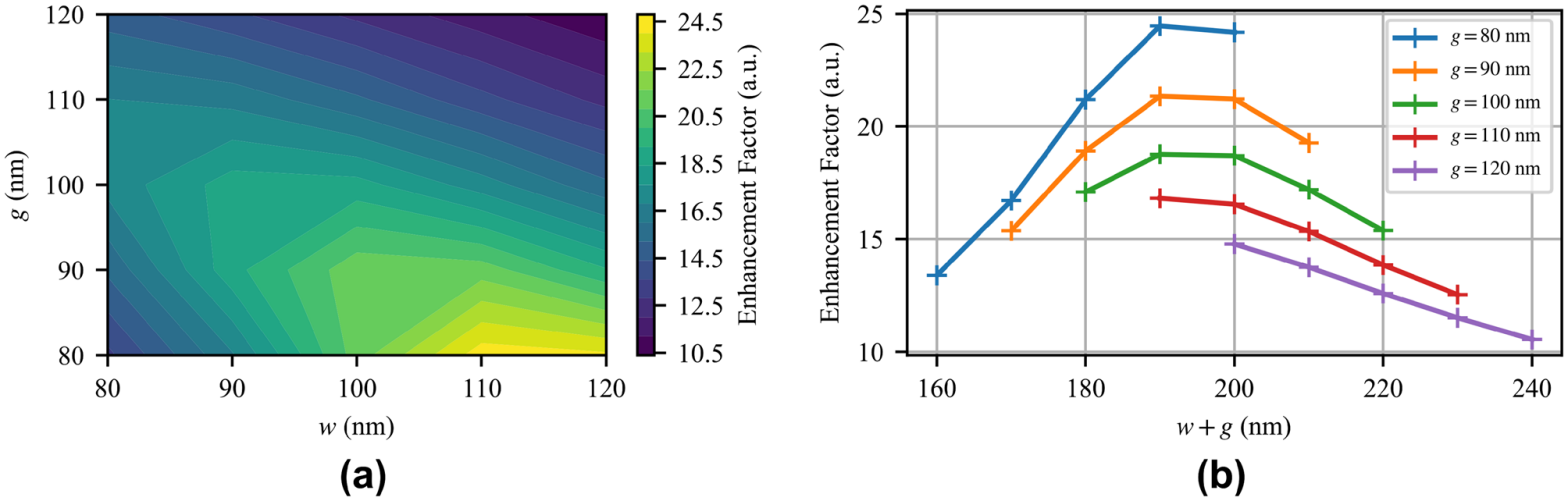
(**a**) Simulated average field intensity enhancement factor, $F_I$, at 15 $\upmu$m (20 THz) with respect to the wire width *w* and the gap width *g*, and (**b**) with respect to the sum of *w* and *g*.

As discussed above, SEIRA demands field enhancement at a targeted wavelength. Maximum field enhancement may occur at the resonance, antiresonance, or somewhere in-between based on material dispersion and coupling efficiency. As a result, targeting maximum reflectance at 15 $\upmu$m (667 cm$^{-1}$, 20 THz) may not lead to ideal sensor performance. To study the field enhancement at 15 $\upmu$m, we simulate the MTM-lined disc with varied gap width (*g*) and wire width (*w*). The study is limited to 80 nm as the minimum feature size due to fabrication constraints and because feature sizes below 80 nm shift the resonance wavelength well below 15 $\upmu$m and the average field enhancement is decreased. Similarly, 120 nm is chosen as the maximum feature size as the resonance shifts as far as 20 $\upmu$m. We once again use $F_I$ to to compare the simulations, and the results are presented in Fig. 3a. The outer radius $b=6(w+g)$ and the pitch $p=2.4b$ are allowed to vary to keep resonance amplitude constant.

In general, decreasing *g* is the most important factor in increasing $F_I$ as the stored electric energy is forced into a smaller volume, which necessarily increases the local electric field intensity. Decreasing the gap size, however, also shifts the resonance wavelength away from 15 $\upmu$m, which reduces $F_I$ at 15 $\upmu$m. The shift in resonance wavelength can then be compensated by a commensurate increase in the wire width *w* or a direct increase in *b* to ensure maximum field enhancement at 15 $\upmu$m. With reference to Fig. 3, the maximum reflection wavelength varies from 13.3 $\upmu$m for smaller feature sizes to 20 $\upmu$m for larger feature sizes, also presented later in Fig. 8b. For gap sizes above 100 nm, the gaps are too large to create a large $F_I$. These data suggest that for a maximum $F_I$, the sum of *w* and *g* approaches approximately 190 nm, as shown in Fig. 3b. Given the fabrication constraints, we fabricate the $(g,w)=(80$ nm, 110 nm) design, which has the best $F_I$ of the cases studied. For these values of *g* and *w*, $b=6(g+w)=1140$ nm and $p=2.4b=2736$ nm.

### Experimental results

Prototypes were fabricated using an EBL lift-off process described in detail in the Methods section. The full EBL pattern consists of eight 200 $\upmu$m $\times$ 200 $\upmu$m arrays of MTM-lined discs, each paired with an equivalent array of solid reference discs of the same thickness, radius and pitch (as with the simulations above). Each array was exposed to an electron beam dosage between 200 and 600 $\upmu$C/cm$^2$ in 50 $\upmu$C/cm$^2$ increments. Arrays with dosages below 450 $\upmu$C/cm$^2$ were underexposed and became underdeveloped, which caused them to lift off entirely in the final sonication step.

**Figure 4:**
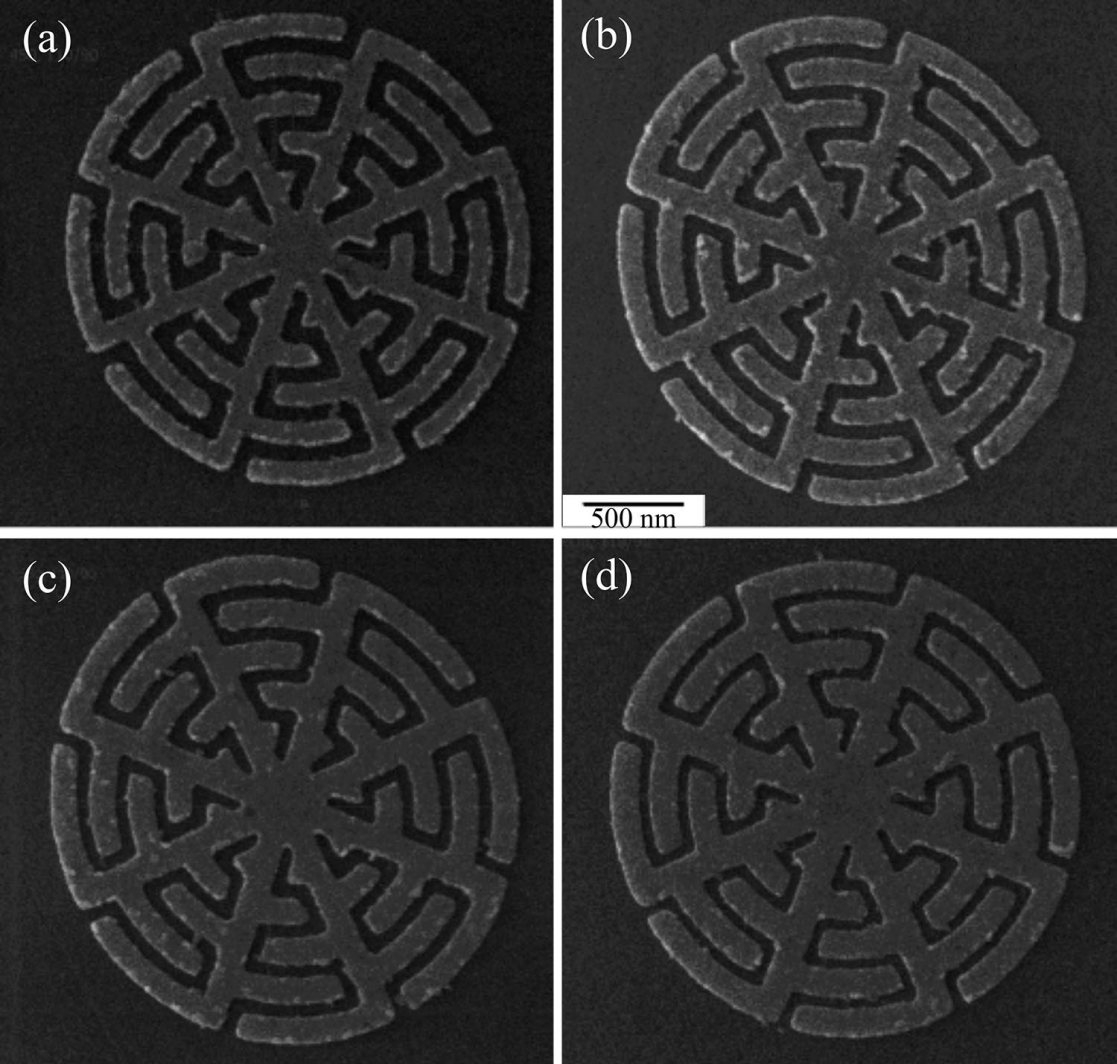
HIM micrographs of patterned MTM-lined discs for dosages (**a**) 450 $\upmu$C/cm$^2$, (**b**) 500 $\upmu$C/cm$^2$, (**c**) 550 $\upmu$C/cm$^2$, and (**d**) 600 $\upmu$C/cm$^2$.

**Figure 5:**
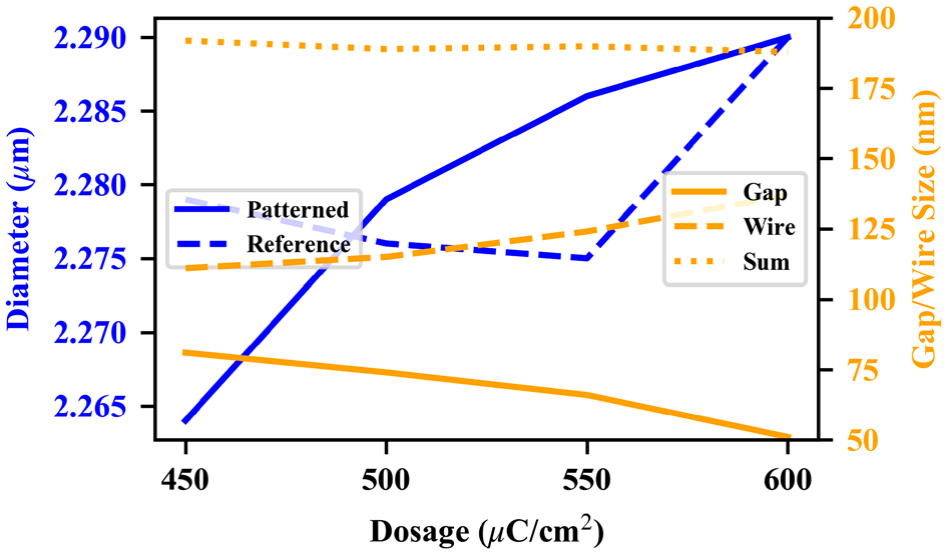
Geometric parameters of MTM-lined disc arrays fabricated by EBL for electron beam dosages of 450–600 $\upmu$C/cm$^2$.

Helium ion micrographs of the fabricated arrays, collected using the Zeiss Orion Helium Ion Microscope (HIM) housed at the University of Alberta nanoFAB Centre, are shown in Fig. 4. Measurements of the unit-cell features are plotted against the EBL dosage in Fig. 5 for the patterned and reference arrays. The pattern with a dosage of 450 $\upmu$C/cm$^2$ approximates the designed feature size best, and also has the highest total sum of feature sizes at 195 nm. Unfortunately, some of the capacitor fingers at this dosage are underdeveloped and those fingers have lifted up during the lift-off process. The sum of the feature sizes is nearly constant over the full dosage range, hence all the fabricated patterns will exhibit strong field enhancement near 15 $\upmu$m; however, the 600 $\upmu$C/cm$^2$ may provide the strongest absorption enhancement due to the smaller gap size as a result of overdosing the wires.

The patterned cell size grows monotonically with the dosage and reaches the designed cell size (diameter$=2280$ nm) at a dosage of 500 $\upmu$C/cm$^2$, and shows a trend towards overdosing at higher dosage values. The reference discs show a less regular trend than the patterned arrays. Differences may be due to difficulties in measuring the disc size through the exact centre of the circle as there is no clear reference, less intricate patterning may have led to a smaller effect of dosage on the overall structure, or difficulties with the sample alignment during the EBL process may have changed the actual dosage delivered to each array.

### Spectroscopy

The FTIR microscope used is limited to frequencies above 600 cm$^{-1}$ due to the decay in the refractive optics. As a result, the full simulated spectrum cannot be observed in the spectroscopic measurements and we have fabricated a shifted, $g=80$ nm/$w=80$ nm set to better visualize the resonance. Additionally, since the eventual measurement of CO$_{2}$ necessitates separating the optical path from the sensing medium, we excite the MTS from the backside of the substrate, as discussed in more detail in the CO$_{2}$ sensing section. To compare such a measurement to simulation, the reflection off the top surface of the substrate is artificially added to the the simulation results. If we assume that the coherence is low and all higher-order reflections off the MTS are captured by the detector, the reflectance becomes a converging geometric series with sum^36^:

1 $$\begin{aligned} R = R_{ZnSe}+\frac{T^2_{ZnSe}R_{MTS}}{1-R_{ZnSe}R_{MTS}}, \end{aligned}$$

where *R* is the reflectance seen in the measured spectrum, $T_{ZnSe}$ and $R_{ZnSe}$ are the normal-incidence reflectance at the air-ZnSe interface calculated by simulation (or equivalently by the Fresnel equations), and $R_{MTS}$ is the normal-incidence reflectance off the ZnSe-MTS/ZnSe-air interface calculated by simulation. Assuming the ZnSe is lossless, $T_{ZnSe}=1-R_{ZnSe}$ and equation 1 becomes:

2 $$\begin{aligned} R = R_{ZnSe}+\frac{(1-R_{ZnSe})^2R_{MTS}}{1-R_{ZnSe}R_{MTS}}. \end{aligned}$$

**Figure 6:**
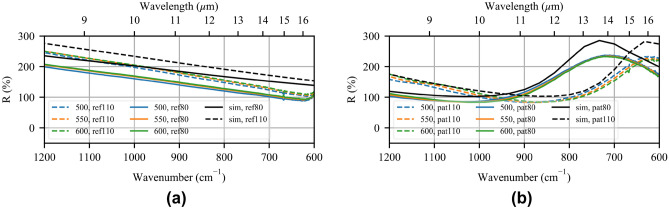
Relative reflection spectra of (**a**) the fabricated reference disc arrays (coloured) and the simulated reference disc array (black), and (**b**) the fabricated MTM-lined disc arrays (coloured) and the simulated MTM-lined disc array (black) normalized to the total reflectance of the bare ZnSe surface (Eq. 3: $R=\sim 0.28$). The dashed curves correspond to the $(g,w)=80/110$ nm case, while the solid curves correspond to the $(g,w)=80/80$ nm case. The spectra have been normalized to remove source and environmental effects.

Reflection spectra for the reference solid disc arrays (labeled “ref”) are shown in Fig. 6a, where the experimental spectra are colored, and the simulation spectra are shown in black. In the absence of a solid gold film to use as a reflectance standard, the total reflectance of the bare ZnSe surface has been used as the background signal to remove environmental absorption. To recreate this standard from simulation data, the reflectance of the MTS can be replaced by the reflectance of ZnSe in Eq. (2):

3 $$\begin{aligned} R = R_{ZnSe}+\frac{(1-R_{ZnSe})^2R_{ZnSe}}{1-R_{ZnSe}^2}. \end{aligned}$$

This normalization (Eq. 3: $R_{ZnSe}=\sim 0.17\rightarrow R=\sim 0.28$) leads to a maximum reflection of $\sim$250% instead of the conventional 100%. The solid curves are the $(g,w)=80/80$ nm case, labeled “80”, while the dashed curves are the $(g,w)=80/110$ nm case, labeled “110”. Only arrays with a successful liftoff procedure are included: 500 $\upmu$C/cm$^2$ in blue, 550 $\upmu$C/cm$^2$ in orange, and 600 $\upmu$C/cm$^2$ in green. The simulated and measured spectra match very well over the plotted range, while the difference in reflection magnitude is due to the dispersion of ZnSe at lower frequencies, the variety of incidence angles present in experiment, and experimental losses not modeled in the simulation, such as scattering from rough surfaces. It is clear that only resonances modeled in simulation are present, and hence any enhancement in reflection is due entirely to the addition of the MTM liner. Few differences are seen when comparing the data for different dosages, which is expected given the size of the discs varies by only 15 nm. The small variations in amplitude are attributed to temperature differences in the cryogenically cooled detector for each measurement. Moreover, the signal amplitude decays in the refractive KBr optics and the sensitivity of the detector is reduced toward lower frequencies (higher wavelengths), which reduces the signal-to-noise ratio of the measurement and the signal decays entirely beyond 500 cm$^{-1}$. No enhanced absorption due to CO$_{2}$ is seen.

Reflection spectra for the patterned MTS arrays, labeled “pat”, are shown in Fig. 6b using the same conventions as before. Once again, the spectra match well over the measured range, with the reflection amplitude significantly increasing near 15 $\upmu$m, as designed. Despite variations in the patterned feature sizes, the reflection of the MTS arrays is nearly unchanging at resonance, showing that the MTM-lined disc array is insensitive to fabrication discrepancies resulting from overdosage. A splitting of the curves is seen towards higher frequencies and is attributed to the geometric variations observed under HIM. The small differences in the spectra outside the resonant band can again be attributed to thermal variations. Furthermore, in these measurements, the predicted absorption at 15 $\upmu$m is dominated by environmental CO$_{2}$ present in the microscope optical path and not absorption enhanced by the MTS, hence no clear absorption enhancement is seen in the normalized spectra.

### CO$_{2}$ sensing

Creating a gas sensor from a SEIRA-enabled surface proves difficult as CO$_{2}$ does not adsorb well to gold surfaces, the molecular density of gases is low compared to liquid and solid analytes, and ambient CO$_{2}$ must be removed from the optical path. Low adsorption may be ideal for a real-time sensor to reduce the time between measured adsorption events, but will inevitably reduce the sensor sensitivity^37^. Functionalization of the gold surface, where a linking molecule is added to chemisorb the target molecule to the gold, is often the preferred solution for increasing the density of excited molecules^5,38,39^; however, such sensors may be single-use or require regeneration cycles. Additionally, the incorporation of high-surface-area materials such as metal-organic framework nanocomposites has been suggested to increase CO$_{2}$ adsorption^40^. Nevertheless, functionalization may be important in the future to improve the sensitivity of the MTM-lined disc MTS to a variety of materials.

**Figure 7:**
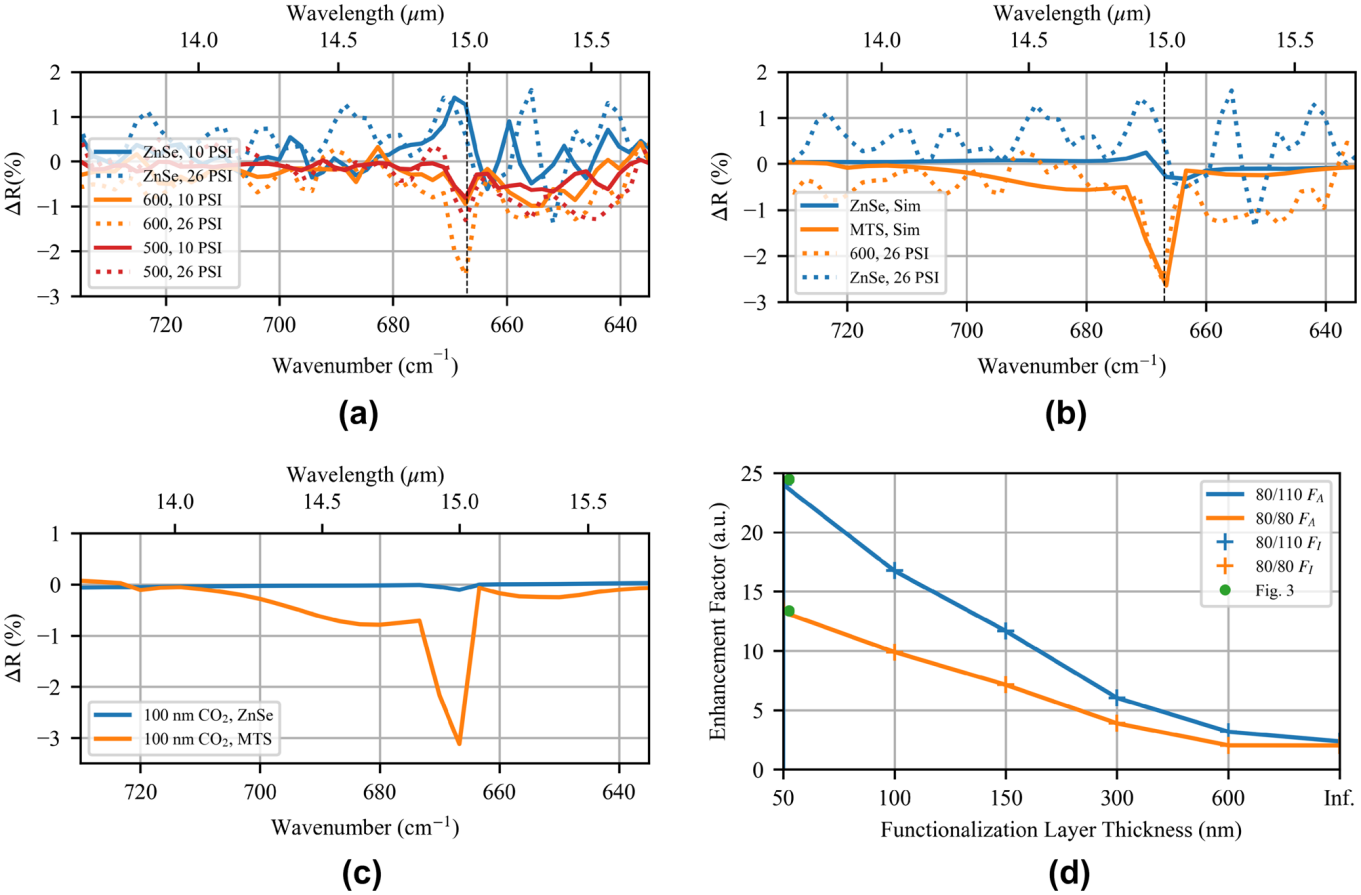
(**a**) Differential MTS reflection data when the gas cell is filled with different pressures of CO$_{2}$ (10 PSI solid, 26 PSI dotted), relative to the case with N$_{2}$ filling. Bare ZnSe is plotted in blue, the case of 600 $\upmu$C/cm$^2$ in orange, and 500 $\upmu$C/cm$^2$ in red. (**b**) Differential reflection data for the 26 PSI case (dotted) plotted against the differential reflection data for a representative simulation with a CO$_{2}$ model (solid). (**c**) Simulated differential reflectance for a 100-nm functionalization layer of CO$_{2}$ at 26 PSI, bare ZnSe vs. MTS. (**d**) Calculated field intensity enhancement factor, $F_I$, and absorption enhancement factor, $F_A$, for the two fabricated cases and various functionalization layer thicknesses. The green dots reproduce the data in Fig. 3, and *Inf.* represents a full half-space of CO$_{2}$, as in the experiment.

Here, we aim to validate the absorption enhancement of physisorbed CO$_{2}$ by the MTM-lined disc MTS, and hence develop a repeatable method of measuring the absorption enhancement for high concentrations of CO$_{2}$. A sealed glass gas cell is designed to support positive pressure and the MTS was fabricated on the inside surface of the gas cell window to improve the adsorption of CO$_{2}$ (see Supplementary Materials for more details). The cell was loaded with either 100% N$_{2}$ or 100% CO$_{2}$ (the gases were not mixed) under variable pressure conditions to increase the adsorption of CO$_{2}$ on the MTS. A far-field, microscope-focused reflection measurement is used and the MTS is excited from the substrate side (see Methods for more details). The relative reflection results of this experiment are plotted in Fig. 7a.

The pressure of both cylinders feeding the gas cell was set to 26 PSI with the exhaust valve closed, and a back-pressure of 10 PSI was measured with the exhaust valve open. For the background case, the gas cell was evacuated of CO$_{2}$ with a continuous flow of N$_{2}$. Any absorption in the baseline is due to incomplete purging of the microscope optical path and its effect is removed by the difference operation. In the solid curves, the reflection is measured under a continuous flow of CO$_{2}$ and an approximate pressure of 10 PSI, measured at the gas cell inlet. Under these conditions, the measured reflection is decreased for the MTS cases (orange and red), while the reflection increases for the bare ZnSe case (in blue), meaning the nanoscale nearfields of the MTSs are able to induce significant absorption by the surrounding CO$_{2}$. When the pressure is increased by a factor of 1.64 to 26 PSI, the differential reflectance improves to $-2.5$% for the 600 $\upmu$C/cm$^2$ case. The 600 $\upmu$C/cm$^2$ case shows the most absorption enhancement due to the smaller gap widths observed in Fig. 4d increasing the overall field enhancement at resonance. Despite wider gaps, the 500 $\upmu$C/cm$^2$ case shows a small absorption enhancement that increases with increasing pressure. Unfortunately, the 550 $\upmu$C/cm$^2$ MTS was destroyed while creating the gas cell.

A representative simulation of the experimental results is shown in Fig. 7b, where the dashed curves are the experimental data and the solid curves are the simulation data. CO$_{2}$ is introduced to the simulation using a line-by-line radiative transfer model to derive the effective optical index for comparison to the experimental results. First, the high-resolution absorption lines derived from the HITRAN database are simulated for 26 PSI^41^. The complex optical index is then calculated from the absorption spectrum using Beer’s law and the Kramers-Krönig relations, and the resolution of the spectrum is reduced to match the experimental resolution by Fourier transform resampling and Happ-Genzel apodization (see Supplementary Materials for the full derivation)^36,42,43^. This resampling method effectively models what occurs in the FTIR, where the ultimate resolution is decided by the length of the interferogram.

A comparison of the simulation to the experimental data suggests an agreement in the trends when the CO$_{2}$ model is applied, particularly at the resonance. A detailed analysis (see Supplementary Materials) of the signal-to-noise ratio shows that the main observed resonance features are significant. For the bare ZnSe case (in blue), the small rise in reflection at 670 cm$^{-1}$ is seen due to an index mismatch at resonance with the addition of the CO$_{2}$ model, while the drop in reflection is due to both absorption and increased transmission. With the presence of the MTS, the resonant evanescent fields are confined very near to the MTS surface and hence absorption is strongly enhanced, both at 667 cm$^{-1}$ and along the band wings. Interestingly, the left band wing is enhanced in simulation, whereas the right band wing is enhanced in the experiment. This is due to the fabrication tolerances marginally shifting the resonance location. In simulation, the contrast enhancement ratio (i.e., $\Delta R_{MTS}/\Delta R_{ZnSe}$ at 667 cm$^{-1}$) is 9.34, much less than was predicted from the intensity enhancement in Fig. 3. If the top-surface reflection is not added, the contrast enhancement ratio decreases further to 6.26.

#### Functionalization layer

To improve the observed contrast enhancement ratio, we consider numerically the case of a thin physical adsorption layer. As discussed above, a functionalization layer that is able to increase the concentration of CO$_{2}$ near the surface of the sample may increase the sensitivity. To model the functionalization layer, the CO$_{2}$ material model is removed from all but the top 100 nm of the sample and replaced with air. In this case, the MTS will continue to strongly enhance the absorption near the surface and contributions to absorption from outside the surface will be suppressed. Conversely, the unenhanced fields near the bare ZnSe will absorb weakly in the thin CO$_{2}$ region, and penetrate far into the lossless air region where they were previously increasing absorption and decreasing the contrast enhancement ratio. As a result, the measurement volume with the MTS could be significantly decreased and the contrast between the MTS and the bare ZnSe surface significantly increased.

The results of this study are shown in Fig. 7c, without adding in the top-surface reflection. For the bare ZnSe surface (in blue), a small reflection differential of $-0.11$% is seen. For the MTS case (in orange), the strong field enhancement within the top 100 nm of the sample continues to enhance the absorption by the CO$_{2}$, and a $-3.11$% dip in reflection is seen at the absorption resonance for a contrast enhancement ratio of 29.2. With the top-surface reflection, the contrast enhancement ratio increases to 33.1, however, the reflection dip decreases to $-1.5$%. The absorption enhancement factor, $F_A$, which is equal to $F_I$ when chemical changes are ignored, is:

4 $$\begin{aligned} F_A = F_I = \frac{1}{V}\int _V\frac{|E_{MTS}|^2}{|E_0|^2}dV = \frac{1}{V}\int _V\frac{Q^{{CO}_{2}}_{MTS}|E_{ZnSe}|^2}{Q^{{CO}_{2}}_{ZnSe}|E_0|^2}dV, \end{aligned}$$

where $F_A$ is the absorption enhancement factor, $F_I$ is the field intensity enhancement factor (Fig. 3), $E_{MTS/ZnSe}$ is the total electric field, $E_0$ is the incident electric field, and $Q^{{CO}_{2}}$ is the power absorbed in the carbon dioxide region *V*. The absorption enhancement factor, $F_A$, is plotted in Fig. 7d at 15 $\upmu$m for varying functionalization layer thicknesses and the two fabricated MTS designs. For a 50-nm layer functionalization layer, the results of Fig. 3 are nearly reproduced (a small variation is present due to the differences between CO$_{2}$ and air). $F_A$ decreases for increasing functionalization film thicknesses until the full transmitted wave is absorbed. The simulations show that in a practical environment, the MTS performs better than $F_A$ suggests as it is $\Delta R^{{CO}_{2}}_{MTS}$ and $\Delta R^{{CO}_{2}}_{ZnSe}$ that are being compared. Reflections off the bare ZnSe surface reduce $|E_{ZnSe}|^2$ below 1 near the surface, suppressing $Q^{{CO}_{2}}_{ZnSe}$. Additionally, more energy is stored within the MTS metal regions due to the small index mismatch between the carbon dioxide and air, and hence increased $Q_{MTS}$ further amplifies the differential reflection amplitude. Due to the 100% CO$_{2}$ concentration used, a meaningful calculation of sensitivity cannot be made.

An additional improvement to the MTS sensor would be to make use of the high permittivity of the ZnSe substrate for attenuated total reflectance (ATR) spectroscopy, exciting the MTS at a grazing angle under total internal reflection conditions and removing the reflection off the top of the substrate^22^. A quantum-cascade laser applied directly to the ZnSe surface would remove interference from atmospheric CO$_{2}$ while significantly improving the signal-to-noise ratio, and air may then be applied to the open surface of the MTS and absorption enhancement may be inferred from the reflected signal. This method allows a stronger excitation of the MTS and is more amenable to integration into other systems. Additionally, if the ZnSe layer is made thin, the beam can be made to excite the MTS several times before proceeding to the detector to increase the interaction time and hence sensitivity of the device. Finally, the experiment could be improved by using mass flow controllers to regulate the gas concentrations inside the cell and increasing the FTIR scan resolution to clearly distinguish the strong absorption peak at 15 $\upmu$m would significantly enhance the minimum amount of CO$_{2}$ detected.

**Figure 8:**
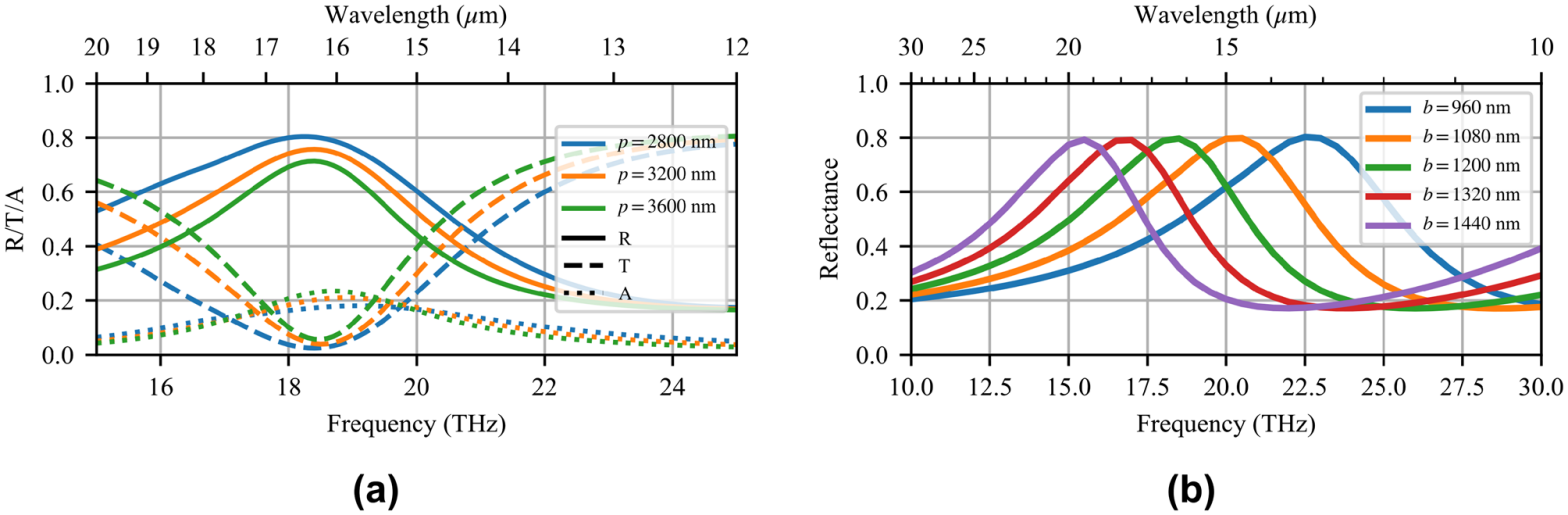
(**a**) Simulated R/T/A spectra for varied MTS pitch *p*, and (**b**) simulated reflection spectra for varied MTS disc radius *b*, while keeping the fill factor constant.

### Resonance tuning

The MTM-lined disc may be tuned through geometric variations to target the absorption bands of other materials. Parametric simulations of the MIR MTM-lined disc reveal many features similar to the NIR MTM-lined aperture^28^. Decreasing the pitch (*p*) of the array leads to increased resonance amplitude as a function of the filling fraction (disc area divided by total unit-cell area), as shown in Fig. 8a. This study does not change the absolute field enhancement, and as a result, the $F_I$ is increased as the filling fraction grows, i.e., as the integrated averaging volume decreases.

Figure 8b shows the simulated reflection spectra for MTM-lined MTSs with varied radius *b*. This sweep is accomplished by setting $w=g=80$–120 nm, $b=6(w+b)$, and $p=2.4b$, which keeps the fill factor constant. These data show that upscaling and downscaling the size of the disc (*b*) respectively redshifts and blueshifts the resonance. The resonance amplitude does not change as the fill factor is held constant for this study. The study presented above for tuning the maximum $F_I$ may also be repurposed to tune the resonant wavelength as the wire and gap widths control a large fraction of the overall unit-cell geometry and are hence extremely important to the MTS performance. With these controls, the resonant band can be tuned over the entire fingerprinting region to target any number of different materials. Importantly, the miniaturization of the elements and the field enhancement is preserved.

## Conclusion

In this paper, we have presented a modified MTM-lined disc for strong average field intensity enhancement in the long-wavelength range of the MIR fingerprint region. The design was created by combining nanoplasmonic gaps with meandered capacitors to create an MTM liner. When properly tuned, this design boasts a greater than four-fold enhancement of average field intensity over a conventional dipole array. Additionally, the MTS is miniaturized, allowing array elements to be closely spaced without loss of field enhancement, and the resonant wavelength may be tuned by changing the MTS element size and/or feature size. The spectra of the fabricated MTSs, designed to enhance absorption by CO$_{2}$ at a wavelength of 15 $\upmu$m, match well with the simulated design spectra. Furthermore, when CO$_{2}$ is passed over the surface, reflection contrast due to absorption is clear. Future developments include functionalizing the MTS to improve adsorption of chemical species, integration of the device with an MIR source, and modulating the beam path to interact more strongly with the MTS.

## Methods

### Numerical methods

The nominal structure was simulated in COMSOL Multiphysics, a full-wave FEM solver, to ascertain the maximum electric field enhancement ratio and to study the effects of the various geometrical parameters on the MTS performance. The simulation region consists of the air input layer, the gold MTS layer (thickness $t=50$ nm), and finally the ZnSe substrate layer, stacked in the *z*-direction. The *x* and *y* boundaries (separated by a distance $p=3$ $\upmu$m) are periodic to simulate an infinite array, and the simulation is excited from the air region with a linearly polarized plane wave. The MTS cell itself is defined by the disc radius $b=1.2$ $\upmu$m, the wire width $w=100$ nm, the gap width $g=100$ nm, and the number of azimuthal cells $N=8$. Each gap is meandered 5 times, and the distance from the last meander to the centre of the cell compensates for changes of the other parameters.

Average field intensity enhancement, $F_I$, is calculated by integrating the field intensity from $z=0$ nm to $z=50$ nm and over the full unit-cell area, divided by the integrated volume.

The equivalent dipole array was simulated in the same fashion, with a *y* pitch of 4.3 $\upmu$m (4.2 $\upmu$m antenna length +50 nm on each end) and an *x* pitch of 9/4.3=$\sim 2.1$ $\upmu$m.

### Electron-beam lithography

ZnSe substrates (ZnSe windows, Edmund Optics) were coated in Electra-92 and a PMMA bilayer at the University of Alberta nanoFAB Centre, then exposed in the JBX-6300FS Electron Beam Lithography System at the University of Waterloo Quantum Nanofab. The samples were then returned to the University of Alberta and the patterns were developed (30 s in 7:3 IPA:H$_{2}$O + 30 s in H$_{2}$O), a 50-nm gold layer was deposited by electron-beam evaporation, and finally lifted off by sonication in acetone.

### Helium ion microscopy

Due to the MTS having features on the order of 100 nm, conventional optical microscopy could not be used to accurately measure the features of the cell. Additionally, the MTS has no continuous conductive path, so scanning electron microscopy cannot be used. Helium ion microscopy is a minimally destructive imaging method with sub-nanometer resolution and the ability to image insulating samples.

The ZnSe chips were affixed to aluminum SEM stubs using carbon tape, then loaded into the Zeiss Orion HIM microscope with Helium and Gallium ion guns. The HIM is also equipped with an electron flood gun to mitigate the positive charge induced by the scattering ions. The images were taken using a current of 1.2 pA with the electron flood gun active and with 64 line-averages.

### FTIR spectroscopy

The reflected spectrum of the array was measured on a Nicolet 8700 Spectrophotometer with a Contiu$\upmu$m microscope attachment. This microscope uses a cryogenically cooled HgCdTe (MCT) detector, a reflective $15\times$ objective, and KBr refractive optics, which limit the acquisition of signals beyond 500 cm$^{-1}$. The microscope is purged with N$_{2}$. The reflection spectrum of each fabricated array was measured over a square 69 $\upmu$m$\times$69 $\upmu$m aperture, and normalized to the reflection spectrum of a bare ZnSe surface.

The Reflachromat lenses used in the Contiu$\upmu$m microscope are symmetric catoptric lenses, and hence none of the components of the focused beam arrive at normal incidence^44^. Furthermore, the Contiu$\upmu$m microscope makes use of a patented aperture system that masks both the input and output beams, rejecting any diffracted power or off-specular reflections^45^. Without exact specifications on the beam path, it is impossible to predict the fractions of the spurious reflected waves (i.e. the top-surface reflection and the higher-order bottom-surface reflections) that reach the detector, and hence we assume in our model that all power is captured, which gives good agreement with the observed experimental data. Due to the normalization, this assumption is accurate as long as the fraction of lost power is similar in both the top reflection and the bottom reflection. Given the wide bandwidth of the source, the coherence is weak and the intensities of the encoded interferograms reflected by all surfaces can be added directly.

### Gas cell

The gas cell consisted of a small, cylindrical glass gas chamber with 1/4” inlets to either side. Gas was fed in from one side and vented from the other. The ZnSe window/substrate was epoxied to the top of the cylinder, and a glass microscope slide was epoxied to the bottom of the cylinder. A more detailed explanation and a figure are available in the Supplementary Materials.

## Supplementary Information

Supplementary Information.
