# Supplementary material for: Surface-enhanced mid-infrared absorption spectroscopy using miniaturized-disc metasurface
Source: Sci Rep. 2021 Dec 7;11:23557. doi: 10.1038/s41598-021-02984-8 (PMC8651662; doi:10.1038/s41598-021-02984-8)
Supplement: Supplementary file 1 — Supplementary Information. [file 41598_2021_2984_MOESM1_ESM.pdf]

Supplementary Materials: Surface-Enhanced Mid-Infrared Absorption Spectroscopy Using Miniaturized-Disc Metasurface

Mitchell Semple[1] and Ashwin K. Iyer[1,*]

[1]University of Alberta, Department of Electrical and Computer Engineering, Edmonton, Alberta, Canada
[*]iyer@ece.ualberta.ca

1 Derivation of CO_2 model

The CO_2 model was derived based on data from the HITRAN database. In summary, spectraplot.com was used to create a high-resolution absorbance spectrum from the HITRAN data for CO_2, then it was converted to an extinction coefficient using Beer's Law. The real optical index was then derived using Kramers-Krönig relations. The high-resolution spectra were resampled separately using the real inverse fast Fourier transform, the apodization procedure used in experiment was applied, and the interferograms were transformed back to the frequency domain.

The HITRAN database is a high-resolution compilation of molecular absorption properties for a wide variety of materials[1]. The absorption spectrum was calculated for a temperature of 300K, 26 PSI, 100% CO_2 molar fraction, a thickness of 100 cm, and frequencies from 580–760 cm^{-1} at a resolution of 0.01 cm^{-1}[2]. The absorbance was then extracted using Beer's law $I = I_0 e^{-\alpha z}$, where I is the output irradiance after travelling through the material layer, I_0 is the input irradiance, α is the absorbance, and z is the thickness of the material layer. Next, the absorbance was related to the extinction coefficient κ_1 using the well-known equation $\alpha = 2\omega\kappa_1/c$, where ω is the radial frequency and $c = 3 \times 10^8$ is the speed of light[3].

Since the complex refractive index is a real, causal, time-invariant spectrum, the real part of the refractive index was derived from the extinction coefficient using the Kramers-Krönig relations[4]:

$$n_1(\omega) - 1 = \frac{2}{\pi} \mathrm{P} \int_0^{\infty} \frac{\omega' \kappa_1(\omega')}{\omega'^2 - \omega} d\omega', \tag{1}$$

where P denotes the Cauchy principal value integral.

With the full complex refractive index, plotted in Fig. S1, the instrument response was modeled next. The instrument response reduces the sample resolution of the modeled spectrum and convolves the spectrum with an apodization function to suppress the sidelobes of resonant peaks. The apodization function used was the Happ-Genzel function[5]:

$$A(x) = 0.54 + 0.46 \cos \frac{\pi x}{X}. \tag{2}$$

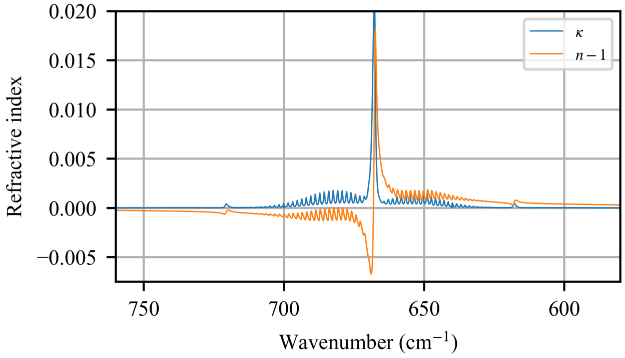

Figure S1: High-resolution complex refractive index model for CO_2.

The FTIR measurement resolution is a function of the maximum path difference inside the interferometer. To model the smaller path difference (and hence lower resolution), the real and imaginary parts of the complex refractive index spectrum were individually inverse fourier cosine-transformed to the spatial domain, truncated at the same path distance of 0.25 cm that was used in measurement, multiplied by the Happ-Genzel apodization function, then Fourier cosine transformed back to the frequency domain:

$$\kappa_2 = \mathcal{F}(A(x)\mathcal{F}^{-1}(\kappa_1)), \tag{3}$$

$$n_2 = \mathcal{F}(A(x)\mathcal{F}^{-1}(n_1)). \tag{4}$$

The resampled and apodized complex refractive index is plotted in Fig. S2, and was directly imported into COMSOL as an interpolated function.

2 Noise Calculations

In the following, noise was calculated on the standard 2100–2200 cm^{-1} region of the spectrum as this region has the strongest signal and only very weak to no interfering absorption bands, depending on the lab environment[6]. Assuming the noise was the same over the measured spectrum, the amplitude of the noise was then extrapolated to the active spectral region near 667 cm^{-1}.

The 2100–2200 cm^{-1} window was first flattened by taking the difference between the raw spectra for N_2 and CO_2. The variance was then calculated on the difference[7]:

$$\sigma_0^2 = \sum_{i=1}^{n} \frac{(y_i - \hat{y})^2}{n-1}. \tag{5}$$

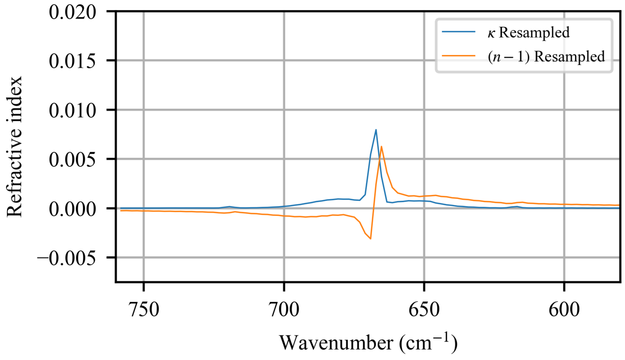

Figure S2: Resampled and apodized complex refractive index model for CO_2.

Next, the variance of the original spectra was calculated. We assumed that the variance calculated above was evenly distributed between the two input spectra and that covariance of the noise was 0, and were hence able to solve for the original absolute variance, σ_1^2, as:

$$\sigma_0^2 = \sigma_1^2 + \sigma_1^2, \tag{6}$$

$$\sigma_1^2 = \frac{\sigma_0^2}{2}. \tag{7}$$

This absolute variance was assumed to be present throughout the entire spectrum. To get the variance on the final presented data, σ_f^2, the relative variance of each spectrum was added in the active region near 667 cm^{-1}:

$$\sigma_f^2 = \left(\frac{A}{B}\right)^2 \left(\frac{\sigma_0^2}{A^2} + \frac{\sigma_0^2}{B^2}\right), \tag{8}$$

where A represents the raw CO_2 data and B represents the raw N_2 data. The final data, plotted with error bars equal to $\pm\sigma_f$, is shown in Fig. S3. The experimental and simulation data agree best for the MTS case, and the ZnSe case would agree better with a constant offset of $\sim$0.5%. Despite the error margins, the dip in reflection seen for the MTS case is clear and lies well outside of the predicted error.

3 Experimental Setup

The experimental setup consisted of the custom glass gas cell loaded onto the Nicolet Continμum microscope stage, connected to gas cylinders and a venting valve via 1/4" plastic tubing. A schematic of the gas cell with illumination is shown in Fig. S4, and the inset shows a cartoon of the MTS

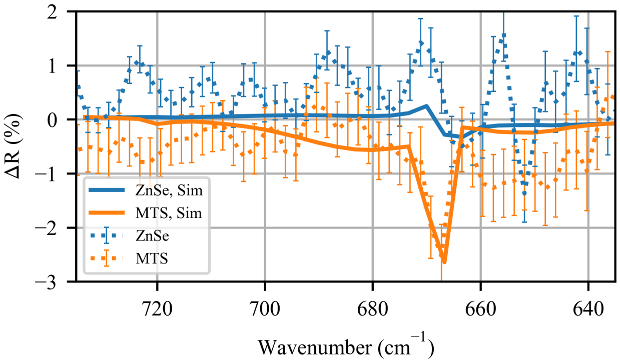

Figure S3: Experimental data with calculated standard error bars (dotted) and simulation data (solid) for comparison.

exciting nearby CO_2 molecules (not to scale). The ZnSe window was epoxied to the glass cell. The microscope was focused onto the bottom surface of the yellow ZnSe substrate, where the MTS is patterned. 100% CO_2 or 100% N_2 gas was fed in from one side, and the pressure was measured with the regulator attached to the gas cell inlet. The output was vented outside the lab through a 1/4" plastic tube. The measurement was prepared by opening the N_2 valve and the venting valve for 2 minutes to purge the gas cell of air. The valves were then closed, the cell was left for 2 minutes to stabilize, and a measurement was taken. The same procedure was used to take measurements with CO_2. The 10-PSI CO_2 measurement was taken with both the CO_2 and the venting valves open. Without mass flow controllers, this setup did not allow precise control over gas mixing nor gas pressure and was chosen as a simple, cheap, but effective means to load the MTS with a known quantity of CO_2.

References

[1] Gordon, I. E. *et al.* The HITRAN2016 molecular spectroscopic database. *J. Quant. Spectrosc. Radiat. Transf.* **203**, 3–69, DOI: 10.1016/j.jqsrt.2017.06.038 (2017).

[2] Goldenstein, C. S., Miller, V. A., Mitchell Spearrin, R. & Strand, C. L. SpectraPlot.com: Integrated spectroscopic modeling of atomic and molecular gases. *J. Quant. Spectrosc. Radiat. Transf.* **200**, 249–257, DOI: 10.1016/J.JQSRT.2017.06.007 (2017).

[3] Hecht, Eugene. *Optics* (Pearson Education Limited, Essex, 2016), 5 edn.

[4] Lucarini, V., Peiponen, K.-E., Saarinen, J. J. & Vartiainen, E. M. *Kramers-Kronig Relations in Optical Materials Research* (Springer-Verlag, 2005).

[5] Griffiths, P. R. & De Haseth, J. A. *Fourier Transform Infrared Spectrometry: Second Edition* (Wiley, 2006).

[6] Boyd, S. & Kansiz, M. Highest Available Signal-To-Noise Performance, Delivering Superior Sensitivity and Analytical Performance. Tech. Rep. SI-01353, Agilent Technologies, Inc. (2011).

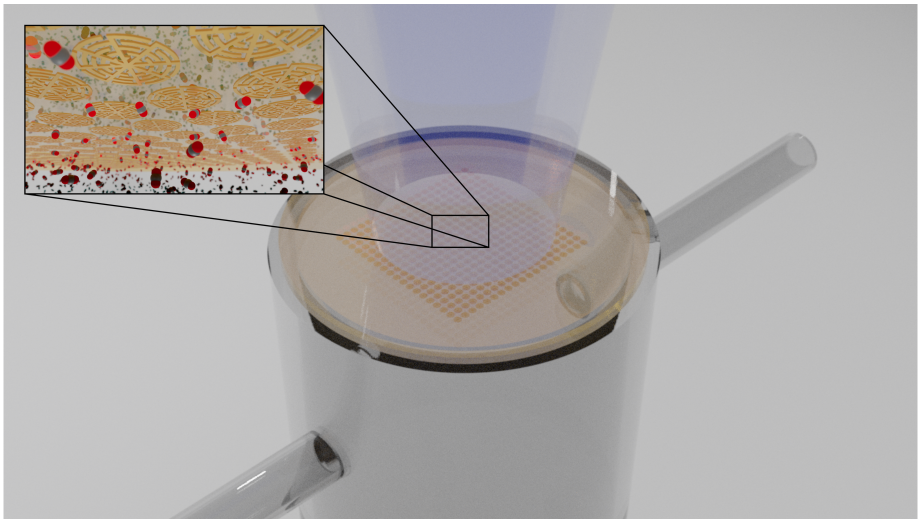

Figure S4: Schematic of the fabricated gas cell, with inset showing a cartoon of the excitation of CO_2 by the MTS (not to scale). The microscope illumination is shown in blue, the ZnSe substrate is shown in yellow with the MTS patterned on the inside surface, and the glass gas cell is clear with two gas inlets.

[7] Ku, H. H. Notes on the Use of Propagation of Error Formulas. *J. Res. Natl. Bureau Standards* 263–273 (1966).
